# The stress phenotype makes cancer cells addicted to CDT2, a substrate receptor of the CRL4 ubiquitin ligase

**DOI:** 10.18632/oncotarget.2042

**Published:** 2014-05-30

**Authors:** Martina Olivero, Daniela Dettori, Sabrina Arena, Davide Zecchin, Erica Lantelme, Maria Flavia Di Renzo

**Affiliations:** ^1^ Department of Oncology, University of Torino, Candiolo, Torino, Italy; ^2^ Candiolo Cancer Institute – FPO IRCCS, Candiolo, Torino, Italy; ^3^ present address: HUGEF, Human Genetics Foundation, Torino, Italy; ^4^ present address: Signal Transduction Laboratory, Cancer Research UK London Research Institute, London U.K.; ^5^ present address: Washington University in St. Louis, St. Louis, MO

**Keywords:** CDT2, Ubiquitin ligase, CDT1, cancer

## Abstract

CDT2/L2DTL/RAMP is one of the substrate receptors of the Cullin Ring Ubiquitin Ligase 4 that targets for ubiquitin mediated degradation a number of substrates, such as CDT1, p21 and CHK1, involved in the regulation of cell cycle and survival. Here we show that CDT2 depletion was alone able to induce the apoptotic death in 12/12 human cancer cell lines from different tissues, regardless of the mutation profile and CDT2 expression level. Cell death was associated to rereplication and to loss of CDT1 degradation. Conversely, CDT2 depletion did not affect non-transformed human cells, such as immortalized kidney, lung and breast cell lines, and primary cultures of endothelial cells and osteoblasts. The ectopic over-expression of an activated oncogene, such as the mutation-activated RAS or the amplified MET in non-transformed immortalized breast cell lines and primary human osteoblasts, respectively, made cells transformed *in vitro*, tumorigenic *in vivo*, and susceptible to CDT2 loss. The widespread effect of CDT2 depletion in different cancer cells suggests that CDT2 is not in a synthetic lethal interaction to a single specific pathway. CDT2 likely is a non-oncogene to which transformed cells become addicted because of their enhanced cellular stress, such as replicative stress and DNA damage.

## INTRODUCTION

The evolutionarily conserved Cullin Ring Ubiquitin Ligase 4 (CRL4) E3 ligase family, together with its DDB1 adaptor, regulates a diverse set of cellular processes including development, transcription, replication and DNA repair [1]. Specificity is conferred by a set of more than fifty substrate receptors, also referred to as DCAFs (DDB1 CUL4 Associated Factors). The CRL4 bound to the substrate receptor CDT2/L2DTL/RAMP (CRL4^CDT2^) promotes the ubiquitylation of proteins in S phase and after DNA damage [2-4]. In vertebrates, the CRL4^CDT2^ targets for destruction the licensing factor CDT1 [2, 3, 5, 6], the CDK inhibitor p21 [7, 8], the histone methyltransferase Set8 [9-11], the histone acetyltransferases GCN5 [12], the checkpoint kinase CHK1 [13] and the TOB anti-proliferative protein [14]. The CRL4^CDT2^ –mediated destruction occurs mostly [2, 15] but not always [13] through its binding to the DNA-bound fraction of the Proliferating Cell Nuclear Antigen (PCNA). The CRL4^CDT2^ has also roles outside the regulation of the cell cycle. For instance, SET8 destruction promotes also transcription and prevents premature chromatin compaction [9, 16]. Moreover, the CRL4^CDT2^ targets the controller of heterochromatin assembly Epe1 [17], the transcription factor E2F in flies [18], the DNA polymerase η in worms [19] and the p12 subunit of the DNA polymerase δ in humans [20, 21], and in fission yeast the ribonucleotide reductase inhibitor Spd1 [22].

CDT2 was first discovered for its ability to induce a transient increase in the proliferation rate of human embryonal carcinoma cells [23]. In most normal adult tissues CDT2 is barely detectable but in highly proliferating tissues, such as testis and bone marrow [23]. CDT2 overexpression was reported in breast [24], gastric [25] and ovarian carcinomas [26] and rhabdomyosarcomas [27] and associated with the aggressiveness of hepatocellular carcinomas [28]. CDT2 overexpression was associated with the gain of 1q where the gene is located [28] in Ewing sarcoma [29] and to the decrease of the miR-30a-5p in primary colorectal carcinomas [30]. Moreover, in diffuse large B-cell lymphoma CDT2 increase might be due to mutation or deletion of the FBXO11 gene, that regulates CDT2 polyubiquitylation and degradation [31, 32].

Thus, we hypothesized that CDT2 could be targeted for cancer therapy and silenced CDT2 in human cancer cell lines and human non-transformed cells. Here we show that CDT2 is necessary for the survival and replication of cancer cells, but dispensable in non-transformed cells.

## RESULTS

### Loss of CDT2 affects viability of cancer cells but not that of non-transformed cells

As we found an association between CDT2 down modulation and the apoptotic death of ovarian cancer cells [33], we investigated if down-modulation of CDT2 with RNA interference was alone able to commit ovarian and other cancer cell lines to death.

CDT2 was suppressed in twelve cancer cell lines from different human tumor tissues and shown to be transformed and tumorigenic, and six non-transformed human cell lines, among which four commercially available cell lines (HK2, hTERT-HME-1, MCF 10A and MRC-5) and two primary cultures of human cells, i.e. Human Umbilical Vein Endothelial Cells (HUVEC) [34] and human osteoblasts (HOB) obtained from cultures of bone-derived cells [35]. These non-transformed cell lines and primary cultures were selected for their expression of CDT, that is detectable only in highly proliferating normal cells [23]. Details of tissues of origin and mutations of the commercially available cell lines are reported in the Supplementary Table 1, which shows that cancer cell lines display different mutation profiles.

CDT2 was silenced in each cell line by means of the transient transfection of a mixture of four small interfering RNAs, each targeting different sequence of the CDT2 mRNA. The use of this siRNA pool allows avoiding too high concentration of each single siRNA and thus prevents off-target effects [36]. On the contrary, pools of siRNA targeting different mRNAs, such as those used in libraries, results in increased off-target effects [37]. As a control, cells were transfected with a non-targeting siRNA pool. Supplementary Figure 1A and 3 and Figure 3 show that these siRNAs were similarly efficient in down-modulating CDT2 in all cell lines, including the non-transformed ones.

**Figure 1:**
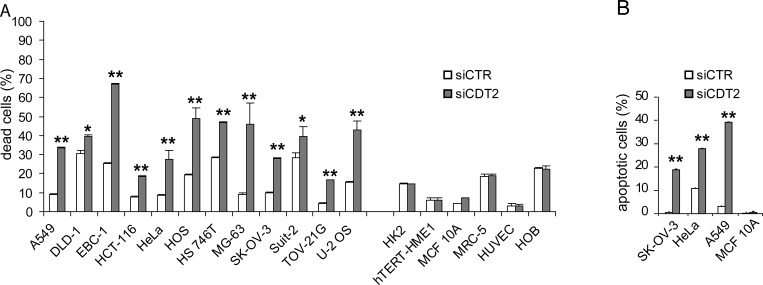
CDT2 suppression affects viability of cancer cells (grouped on the left), but not that of non-transformed cells (grouped on the right) Cell lines were transfected with either the CDT2 specific (siCDT2) or a control (siCTR) small interfering RNA pool. (A) Percentage of dead cells, measured with cytometry after cell labeling with propidium iodide: the non-transformed cell lines and primary cell cultures (on the right) were similarly not affected by CDT2 silencing, which induced the death of all cancer cell lines (on the left). (B) Percentage of apoptotic cells, measured with cytometry after cell labeling with an anti active caspase-3 antibody. In cancer cell lines (SK-OV-3, HeLa and A549) the number of caspase-3 positive cells increased after CDT2 silencing that did not affect the non-transformed cell line MCF 10A. Significance was evaluated using the Student's t-test: ** *P*<0.01, *P<0.05.

Depletion of CDT2 committed to death only cancer cell lines (Figure 1A). An increased number of active caspase-3-positive cells after CDT2 silencing was observed in cancer cell lines, but not in non-transformed cells (Figure 1B). These data show that CDT2 depletion resulted in decreased viability of cancer cell lines, due to apoptosis activation. As shown in the Supplementary Figure 1B, no correlation was found between cell susceptibility to CDT2 silencing and the baseline level of CDT2 expression. It is noteworthy that all the cancer cell lines were susceptible to CDT2 silencing and all the non-transformed cells showed resistance, although in all lines a comparable protein silencing was achieved (Supplementary Figure 1A). To further confirm the specific effect of CDT2 silencing, both transformed and non-transformed cells were transduced to express shRNAs targeting a different CDT2 sequence. Supplementary Figure 2 shows that transformed cells, but not non-transformed cells were selectively killed by CDT2 silencing.

### Loss of CDT2 causes rereplication in cancer cells, but not in non-transformed cells

It has been shown [5, 7] that CDT2 depletion might cause rereplication and G2/M arrest in cells.

**Figure 2:**
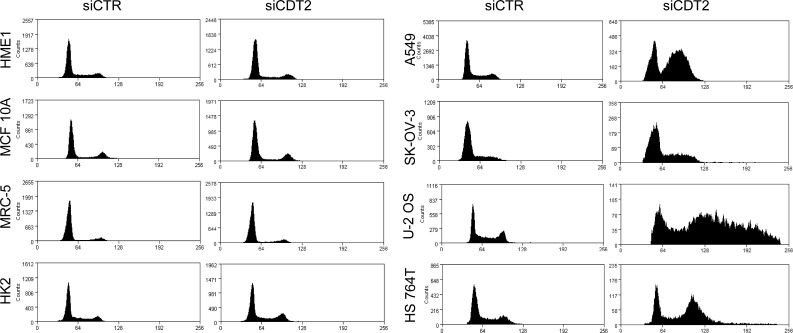
CDT2 suppression induces rereplication and cell cycle blockade in cancer cells, but not in non-transformed cells Analysis of the cell cycles of cancer (right panels) and non-transformed (left panels) cells after transfection with either the CDT2 specific (siCDT2) or control (siCTR) small interfering RNA pool. Only in cancer cells CDT2 silencing resulted in alterations of the cell cycle.

We evaluated the effect of CDT2 silencing on the cell cycle (Table 1 and Figure 2). All cancer cell lines showed an altered cell cycle after CDT2 suppression, while the cell cycle of non-transformed cells was not affected (Table 1 and Figure 2). In cancer cell lines, the number of cells in G0/G1 phase was strikingly reduced. Moreover, accrual of cells in G2/M and increased number of cells showing rereplication, i.e. cells showing DNA content >G2/M (>4N), were both associated to 72 hours long CDT2 depletion (Table 1). To better characterize the cell cycle blockade, we examined the status of histone H3 Ser10-phosphorylation, that is nearly absent in interphase cells and occurs almost exclusively during mitosis [38]; histone H3 phosphorylation increases in proliferating cells and its decrease is indicative of lack of entry into mitosis. As shown in Supplementary Figure 2, after CDT2 silencing phospho-H3 positive cells diminished in cancer cell lines, but not in non-transformed cell lines. These data show that CDT2 silencing induced cell cycle blockade, G2 arrest and rereplication in cancer cells, but not in non-transformed cells.

**Table 1:** Cell cycles of cancer and non-transformed (non tumorigenic) cell lines after CDT2 silencing The percentage of cells in each phase of the cell cycle is shown. Cell cycle analysis was carried out by labelling cells with propidium iodide (PI) after 72 hour long CDT2 silencing and evaluating the PI content in each cell with cytometer

Cell line	tumorigenicity	G0/G1		S		G2/M		>G2/M	
		siCTR	siCDT2	siCTR	siCDT2	siCTR	siCDT2	siCTR	siCDT2
A549	YES	63	14	22	41	13	24	2	21
DLD1	YES	47	24	19	24	26	27	8	25
EBC1	YES	45	30	34	30	16	26	5	14
HCT116	YES	39	24	31	25	24	20	6	31
HeLa	YES	40	21	21	14	25	24	14	41
HOS	YES	42	28	16	16	37	39	5	17
HS764T	YES	45	18	30	26	22	25	3	31
MG63	YES	61	30	13	9	23	43	3	18
SK-OV-3	YES	67	45	19	20	12	20	2	15
SUIT2	YES	59	23	25	39	13	28	3	10
TOV-21G	YES	50	26	22	37	26	25	2	12
U2OS	YES	43	10	20	9	33	21	4	60
HK2	NO	65	58	19	19	15	21	1	2
hTERT-HME-1	NO	72	73	15	15	11	10	2	2
HOB	NO	75	72	14	13	10	13	1	2
HUVEC	NO	63	57	14	16	20	24	3	3
MCF 10A	NO	79	78	8	9	12	12	1	1

### Loss of CDT2 affects the degradation of CDT1 in cancer cells, but not in non-transformed cells

**Figure 3:**
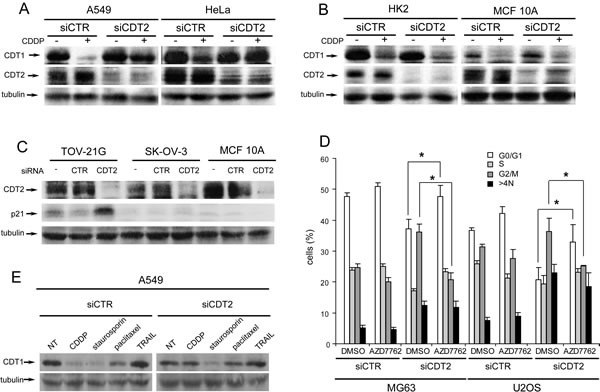
Loss of CDT2 affects the degradation of CDT1 in cancer, but not in non-transformed cells (A-B), Western blot analysis of CDT1 and CDT2 proteins in cancer (A) and non-transformed (B) cell lines transfected with either CDT2 specific (siCDT2) or control (siCTR) small interfering RNA pool and treated with 10 μM CDDP for 6 hours. (C) Western blot analysis of p21 in p53 defective cancer cells (SK-OV-3) and in p53 proficient cancer (TOV-21G) and non-transformed (MCF 10A) cell lines, transfected as above. (D) Cytometric analysis of cell cycles of MG63 and U2OS cancer cell lines transfected with siRNA as above for 48 hours and then incubated in medium containing dimethylsulphoxide (DMSO) or 30nM AZD7762 CHK1 specific inhibitor for 24 hours. (E) Western blot analysis of CDT1 in A549 cancer cells, transfected with control and CDT2 specific siRNAs as in panels A and B and treated with CDDP (10 μM), staurosporin (1 μM), paclitaxel (10 nM) or TRAIL (100 μg/ml) for 6 hours. Significance was evaluated using the Student's t-test: *P<0.05.

As all cancer cell lines underwent rereplication after CDT2 depletion, we inferred that the stability of the licencing factor CDT1 was affected. As expected, cell treatment with the DNA damaging agent cisplatin (CDDP) resulted in CDT1 degradation in both cancer and non-transformed cells transfected with control siRNAs (Figure 3A-B). Conversely, CDT2 silencing abrogated the CDDP induced degradation of CDT1 in cancer cells, while it did not affect CDT1 degradation in non-transformed cells (Figure 3A-B). In agreement, it has been shown already that increased level of CDT1 results in rereplication and cell apoptotic death [39]

We assessed also the possible role of either p21 or CHK1, that are also CRL4^CDT2^ substrates. As shown in Figure 3C, p21 degradation was impaired after CDT2 depletion, but only in p53 proficient cancer cell lines (such as TOV-21G) and not in p53 defective cancer cell lines (such as SK-OV-3) and in p53 proficient non-transformed cell lines (such as MCF-10A).

The role of CDT2 in the degradation of CHK1 in stressed cells has been already shown [13]. The inhibition of CHK1 kinase activity in CDT2 depleted cells resulted only in lack of G2/M arrest but did not change rereplication (Figure 3D).

To discriminate whether CDT2 was recruited and necessary in cancer cells because of the DNA damage or because of the apoptotic stimulus, CDT2-depleted cancer cells were treated with proapoptotic agents that do not induce DNA damage such as paclitaxel, TRAIL and staurosporin. Figure 3E shows that CDT2 depletion did not affect CDT1 degradation that occurred in response to the non-DNA-damaging agents.

In conclusion, these data demonstrate that CDT2 is indispensable for CDT1 degradation in response to DNA damage only in cancer cells.

### DNA rereplication causes the death of CDT2 depleted cells

**Figure 4:**
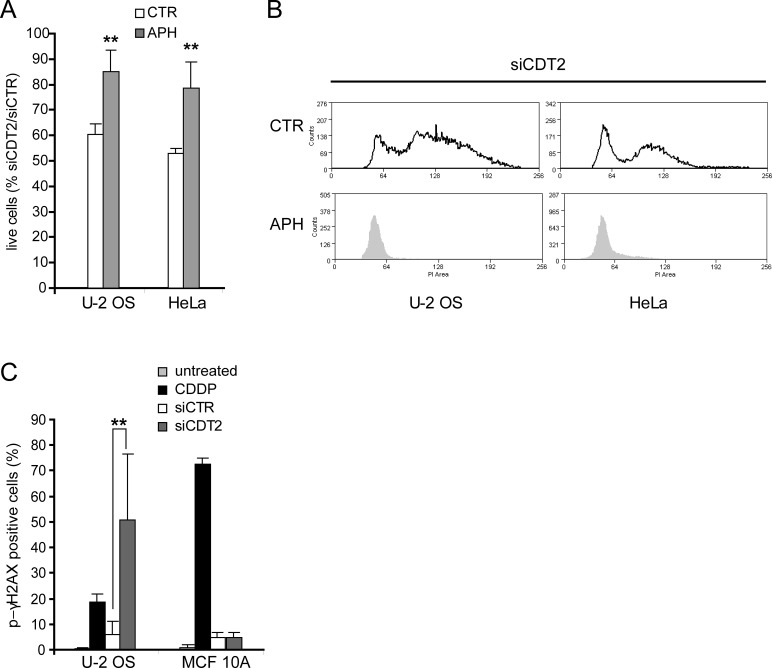
DNA rereplication causes the death of CDT2 depleted cells (A) Percentage of live cells (PI neg and Annexin V negative) after cell transfection with the CDT2 specific or control siRNAs, and after cell treatment with aphidicolin (APH, 0,25μg/ml) or control medium with DMSO (CTR) for 48 hours. Graphs show that cell treatment with aphidicolin reduced the proapoptotic effect of CDT2 depletion. (B) Cycles of cells transfected as above and treated with aphidicolin (APH) or control medium with DMSO (CTR) as in panel A; cell cycle analysis shows that both U-2 OS and HeLa cells were blocked at the G1 phase by the treatment with aphidicolin and did not undergo rereplication. (C) Percentage of p-γH2AX positive cells in response to CDT2 depletion. Both cancer (U2-OS) and non-transformed (MCF 10A) cells, transfected with control and CDT2 specific siRNAs were labeled with an antibody directed against the phosphorylated form of γH2AX (Ser10). Labelled cells were measured using cytometry. Only in cancer cells CDT2 depletion resulted in an increase of p-γH2AX positive cells similar to that caused in both cancer and normal cells by CDDP. In panels A and C significance was evaluated using the Student's t-test: ** *P*<0.01.

To confirm the correlation between CDT2 depletion, rereplication and death we used the DNA replication inhibitor aphidicolin, which blocks cells at the G1/S transition [40]. In cancer cell lines, cell treatment with aphidicolin resulted in better cell survival after CDT2 silencing (Figure 4A) and blocked rereplication (Figure 4B). This shows that, after blocking the cell cycle, cancer cells were no longer able to undergo DNA replication and rereplication after CDT2 depletion. Reduced rereplication resulted in increased cell survival.

Cell death follows rereplication likely because rereplication causes DNA damage [41]. We measured the phosphorylated γH2AX histone in CDT2 depleted and control cells, as its phosphorylation is a reporter of double strand breaks (DBS) in DNA [42]. As a control, we compared cells treated with CDDP to untreated cells, as DNA damaging agents induce γH2AX phosphorylation. Both cancer and non-transformed cells treated with CDDP showed an increased phosphorylation in γH2AX (Figure 4C). Figure 4C also shows that CDT2 depletion alone caused a similar effect in cancer cells, indicative of DNA damage, but not in non-transformed cells.

### The transformed phenotype makes cancer cells addicted to CDT2

We have shown above that several cancer cell lines, with different mutation profiles, underwent rereplication after CDT2 suppression. We thus hypothesized that CDT2 depletion is indispensable in cancer cells because of their basal stress phenotype associated to transformation, due to DNA damage, DNA replication stress and mitotic stress [43]. To understand whether the transformed phenotype makes cancer cells “addicted” to CDT2, we converted non-transformed cells into transformed and tumorigenic cells and silenced CDT2. Using Lentiviral vectors, we transduced the non-transformed, spontaneously immortalized MCF 10A and the h-TERT immortalized HME-1 breast epithelial cells to over-express the RAS oncogene activated by the p-Gly13Asp mutation (referred as KRAS G13D) (Supplementary Figure 3A and B). It was shown previously shown that both MCF 10A and h-TERT-HME-1 expressing KRAS are transformed *in vitro*, i.e. able to grow in soft agar medium, and tumorigenic *in vivo*, i.e. able to form tumors when xenografted in immunocompromised mice [44, 45]. Moreover, the primary HOBs were transduced to overexpress the MET oncogene (Supplementary Figure 3C); these MET overexpressing HOB cells were previously shown to be transformed and tumorigenic [35].

**Figure 5:**
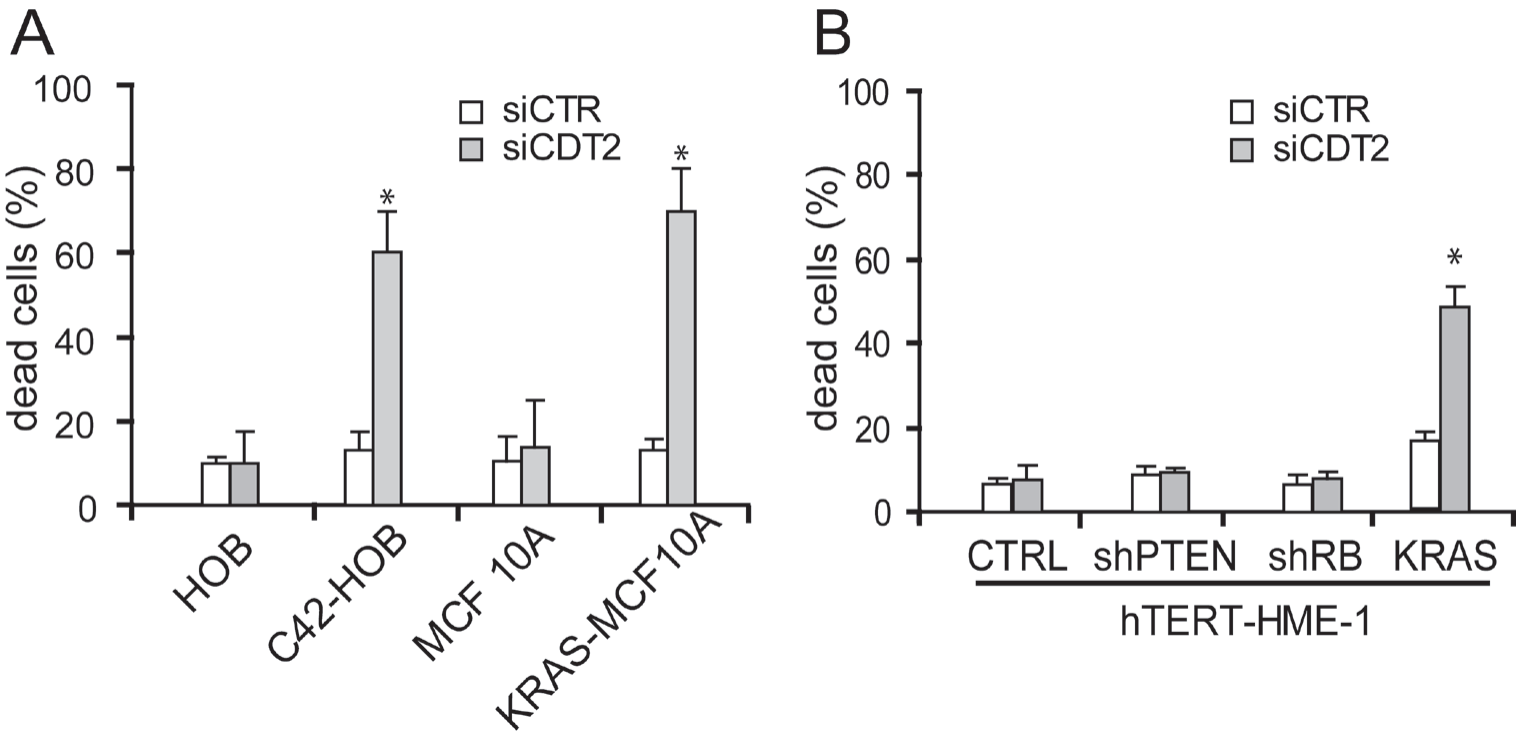
Expression of an activated oncogene makes cells addicted to CDT2 The indicated cell populations were transfected with either the CDT2 specific (siCDT2) or a control (siCTR) small interfering RNA pool. Percentage of dead cells, measured with cytometry after cell labeling with propidium iodide, is shown in both panels. (A) The non-transformed MCF 10A breast cells and primary cultures of human osteoblasts (HOBs) were transduced to express the activated KRAS oncogene (KRAS-MCF 10A) and to over-express the MET oncogene (C42-HOB), respectively (see also Supplementary Figure 3). Both the KRAS-MCF 10A and the C42-HOB died after CDT2 silencing, while the parental cells were unaffected. (B) The non-transformed hTERT-HME-1 breast cells were transduced either to express the activated KRAS oncogene or to express the PTEN or RB1 specific shRNA. Only the KRAS expressing hTERT-HME-1 were killed by CDT2 silencing. Significance was evaluated using the Student's t-test: * *P*<0.05.

As shown also above, the non–transformed cells were not affected by CDT2 suppression (Figure 5), while the expression of the hyperactivated oncogenes up-regulated CDT2 expression (Supplementary Figure 3) and rendered cells susceptible to CDT2 loss (Figure 5A and 5B).

Conversely, the hTERT-HME-1 cells where the tumor suppressor genes RB1 or PTEN were knocked-down by means of specific shRNA did not acquire a transformed and tumorigenic phenotype [45] and were insensitive to CDT2 loss (Figure 5B). Notably, the parental recipient hTERT-HME-1 cells carry already a functionally inactive TP53, because of the p.Cys176Phe mutation [45], that is one of the most frequent TP53 mutation and has been detected in multiple tumor types.

Altogether these data show that the acquisition of a transformed phenotype makes cells susceptible to CDT2 loss.

## DISCUSSION

This work shows that CDT2 is indispensable for the survival of cancer cells, but not of non-transformed cells. Cancer cell death due to CDT2 depletion was caused by rereplication.

CDT2 is the substrate receptor of the CRL4^CDT2^ ubiquitin ligase complex, which targets for destruction a number of substrates, among which the licensing factor CDT1 [2, 3, 5, 6], the CDK inhibitor p21 [7, 8], the checkpoint kinase CHK1 [13] and the p12 subunit of the DNA polymerase δ [20, 21]. CDT1 is a component of the prereplication complex that should be disassembled once the DNA synthesis begins. The activity of CDT1 during the cell cycle is tightly regulated by its association with the protein geminin and by its targeting for destruction by different ubiquitin ligase. Upon DNA damage, CDT1 is rapidly targeted for degradation by the CRL4^CDT2^ complex. This targeting safeguards genomic integrity and prevents rereplication while DNA repair is in progress. Indeed, it has been shown already that accumulated CDT1, because of reduced degradation results in DNA rereplication and cell apoptosis [39]. It has been also shown that CDT1 destruction occurs in cells after treatment with chemotherapeutics [46] and after UV irradiation [47, 48]. We report here that in cancer cells, but not in non-transformed cells, this mechanism was consistently impaired by CDT2 depletion. P21 is also a substrate of the CRL4^CDT2^ ubiquitin ligase. However, its involvement in the DNA rereplication and death of the twelve cancer cell lines studied here is unlikely as most of these lines are p53 defective and thus generally unable to respond to DNA damaging agents and apoptotic stimuli by increasing p21 expression. Indeed, we found that p21 degradation is impaired after CDT2 depletion only in p53 proficient cancer cell lines. In case of replication stress, lack of degradation of the checkpoint protein kinase CHK1 occurs in CDT2 depleted cancer cells [13]. We found that CDT2 depleted cancer cells accumulated in G2/M. However, rereplication and cell death do not depend on CHK1 reduced degradation, as cells in the presence of CHK1 inhibitor did not accumulate in G2/M but underwent rereplication anyway. On the other side, it is not surprising that CDT2 depleted cells arrested in G2/M showed rereplicated DNA, because CDT1 degradation was no longer feasible and G2/M arrest enhances rereplication [49]. Moreover, CDT2 depleted likely accumulated the p12 subunit of the DNA polymerase δ, that allow fork progression after DNA damage [21].

It has been reported that the suppression of geminin, which is the specific protein inhibitor of CDT1, induces cell death in some, but not all, cancer cell lines, and also in some non-transformed cells, by inducing rereplication and activating the DNA damage checkpoint [50, 51]. We show here that CDT2 depletion resulted effective in killing cancer cells that are unaffected by geminin depletion [50]. One possible explanation is that CDT2 loss also results in lack of CHK1 degradation and cell accrual in G2/M, which as mentioned above enhances rereplication [49].

Rereplication was likely the cause of the death of CDT2-depleted cancer cells. Cell treatment with aphidicolin, which blocks cells at the G1/S transition [40] blocked rereplication and improved cell viability after CDT2 silencing. After CDT2 suppression cancer cells underwent an apoptotic type of death, as shown by the accumulation of active caspase-3. This is in agreement with the finding that rereplication generates single strand and double strand DNA breaks [52]. Indeed, we show that rereplication was associated to the increase of phosphorylated γH2AX histone that is the marker of double strand breaks in DNA. These data are in agreement with the finding of increased phosphorylation of γH2AX histone in two cancer cell lines where components of the CRL4^CDT2^ complex had been silenced [53]. It has been also shown that CDT2 depletion causes the phosphorylation, i.e. activation, of CHK1 [54] that follows generation of single strand DNA. Therefore, it is likely that damage-responsive pathways sense rereplication as DNA damage [41] and eventually trigger the apoptotic machinery if the DNA damage repair pathways are not functioning, as in cancer cells.

We show here that cell death, rereplication and CDT1 accumulation after CDT2 depletion occurred exclusively in cancer cells. This finding is important: if DNA rereplication and cell death could be induced selectively in cancer cells by CDT2 depletion, cancer cells could be killed without harming normal cells. The resistance of non-transformed cells to CDT2 depletion was unexpected, as homozygous Cdt2^−/−^ mouse embryos die at the two- to four-cell stage with an abnormal nuclear morphology [55]. This implies that in normal cells CRL4^CDT2^ is dispensable and one or more alternate mechanisms of CDT1 degradation were actively functioning while in cancer cells CRL4^CDT2^ is indispensable. It is worth noting that although several mechanisms regulate CDT1 degradation, all cancer cell lines shown here were similarly susceptible to CDT2 loss, though they are characterized by different pattern of mutations. This wide effect of CDT2 depletion in suggests that CDT2 in not in a synthetic lethal relationship to another specific gene or pathway but becomes dominant in cancer cells.

In agreement, the addition of a hyperactivated oncogene made transformed and tumorigenic the non-transformed cells and was alone sufficient to make them dependent on CDT2. Thus, we propose that the spontaneously occurring and the ectopically obtained transformed cells are more vulnerable to the loss of CDT2, because they are in constant need of CRL4^CDT2^. This necessity might be associated to their stress phenotype, due to DNA damage, replication stress and mitotic stress [43]. Notably, the CRL4^CDT2^ complex is distinctively recruited for CDT1 degradation after DNA damage. The compound pattern of genetic alterations in cancer cells leads to a constitutive level of endogenous stimuli, which indeed results in activation of the response to DNA damage and replication stress [56]. Although altered, however, even cancer cells should save mechanisms that ensure the maintenance of cell replication. This is likely held by the same mechanisms that protect replication in normal cells.

In line, CDT2 expression is elevated in almost all the cancer cell lines studied here, as it is in several other cancer cell lines and human cancer samples [24, 25, 27-29]. This can be correlated to the increased proliferation rate of cancer cells versus normal cells, as the non-transformed highly proliferating tissues such as testis and bone marrow show an elevated level of CDT2 expression [23]. Moreover, it has been already demonstrated that replication stress, that could be constitutively increased in cancer cells, determines increased expression of CDT2 in both mammalian cells [13] and fission yeast [22]. The exquisite sensitivity of cancer cells to CDT2 loss may explain, in part, why CDT2 overexpression is positively selected during tumorigenesis, as it might provide cancer cells with a selective advantage.

Interestingly, aspects of CRL4^CDT2^ loss are phenocopied by cell treatment with MLN4924 [57], a small molecule that inhibits the CRL-NEDD8-activating enzyme (NAE), which is also effective in actively proliferating non-transformed cells. This is expected as conjugation of the ubiquitin-like protein NEDD8 is required to activate all the CRL ligases. Thus, inhibiting the NAE by MLN4924 prevents destruction of numerous substrates of the CRLs, involved in cell proliferation and cancer pathways, such as not only CDT1 and p21, but also cyclins and checkpoint kinases [26, 39, 57, 58]. Therefore, treatment of cancer cells with MLN4924 triggers rereplication, DNA damage, G2 arrest, and apoptosis. MLN4924 is currently in clinical trials as an anticancer agent [59].

Altogether, data shown here suggest that cancer cells share some properties that make them “addicted” to CDT2. The term “oncogene addiction” [60] has been invented to pinpoint the dependence of cancer cells on a mutated cancer gene for tumor initiation and maintenance. One facet of oncogene addiction is synthetic lethality, as cancer cells might become addicted to a given oncogene when they lose a redundant gene or pathway that is in synthetic lethal interaction with the oncogenic pathway [61, 62]. Moreover, activation of an oncogene or loss of a tumor suppressor gene might install a flood of genetic, transcriptional and metabolic alterations [63-66] that make the cancer gene the only “dam to the flood” of pro-apoptotic signals [67]. Therefore, despite the focus on causative oncogenes as targets of cancer therapeutics, there is solid experimental evidence for non-oncogenes that are rate-limiting to their pathways and represent potential drug targets. This phenomenon has been termed “non-oncogene addiction” in reference to the increased dependence of cancer cells on the normal cellular functions of certain genes, which themselves are not classical oncogenes [43]. Here we show that several cancer cell lines, which derive from diverse tissues and show different genetic alterations, all require CDT2 for proliferation and survival and are thus addicted to this non-oncogene. We can infer that CDT2 is in synthetic lethal interaction with different oncogenic pathway, the most likely Achille's heel being the stress phenotype of cancer cells, due to the DNA damage represented by the widespread genetic aberration and the replication and mitotic stress caused by uncontrolled proliferation.

## METHODS

### Cell lines, chemicals and antibodies

Twelve cancer cell lines (Table S1) from different human tumors and the non-transformed human cell lines HK2, hTERT-HME1, MCF 10A and MRC-5 cell lines were purchased from the American Type Culture Collection (Manassas, VA) and grown as suggested by the provider. Primary cultures of human cells HUVEC and osteoblasts (HOB) were obtained as previously described [34, 35]. MCF 10A and hTERT-HME-1 cells were engineered to overexpress the KRAS G13D cDNA by infection with a Lentiviral vector harboring the mutated KRAS allele downstream a constitutive promoter as previously reported [45]. C42 MET over-expressing HOB clone were obtained as previously reported [35]. Details of other reagents are described in Supplementary Materials and Methods.

### RNA Interference

RNAi experiments were performed using ON-TARGET *plus* SMART pool, a mixture of four siRNAs targeting one gene (Dharmacon, Lafayette,CO). In each experiment ON-TARGET *plus* Non-Targeting Pool (Dharmacon, Lafayette, CO) was used as negative control. The sequences of the oligonucleotides are reported in Supplementary Materials and Methods. Cell lines were plated at 30-40% confluence and transfected with the indicated siRNA pools (200nM) using Oligofectamine (Invitrogen, Eugene, OR) according to the manufacturer's instructions. The mRNA downmodulation of target genes was assayed with quantitative RT-PCR and with Western Blot analysis 48h and 72h after transfection, respectively. Experiments were performed 72 hours after transfection, if not otherwise indicated.

### RNA extraction and Quantitative Reverse Transcription-PCR

RNA extraction and qPCR was carried out as described previously [33]. Details are reported in Supplementary Materials and Methods.

### Protein extraction and Western Blot analysis

Total protein extraction was performed by directly incubating cells in SDS containing lysis buffer at 95°C for 5 minutes. Proteins were separated by PAGE and transferred to nitrocellulose sheets. Equal amounts of proteins (100 μg) were loaded in each lane. Blots were probed and when necessary re-probed with the different antibodies as indicated in the Result section. Bound antibodies were detected using the appropriate peroxidase-conjugated secondary antibody and revealed by Enhanced Chemiluminescence (Amersham, United Kingdom).

### Flow cytometry analysis

Cell cycle analysis was based on DNA content. Details are reported in Supplementary Materials and Methods.

## SUPPLEMENTARY MATERIALS AND METHODS TABLE AND FIGURES


