# Supplementary material for: The stress phenotype makes cancer cells addicted to CDT2, a substrate receptor of the CRL4 ubiquitin ligase
Source: Oncotarget. 2014 May 30;5(15):5992–6002. doi: 10.18632/oncotarget.2042 (PMC4171607; doi:10.18632/oncotarget.2042)
Supplement: Supplementary file 1 [file oncotarget-05-5992-s001.pdf]

The stress phenotype makes cancer cells addicted to CDT2, a substrate receptor of the CRL4 ubiquitin ligase

Supplementary Materials and Methods

Chemicals and antibodies. Cisplatin (CDDP) was from Bristol-Myers Squibb (Rocky Hill, NJ). Staurosporine, taxolo e trail were from Sigma (Steinheim, Germany). Aphidicolin was distributed by Sigma-Aldrich (Saint Louis, MO). AZD7762 was distributed by Selleck Chemicals. Anti-CDT2 and anti-CDT1 antibody produced in rabbit was distributed by Bethyl Laboratories (Montgomery, TX). Purified rabbit anti-active Caspase 3 monoclonal Ab was distributed by BD Pharmingen (San Diego, CA). Annexin V-APC was distributed by Bender MedSystems GmbH (Wien, Austria). Anti-phosho-Histone H3 (Ser10) was distributed by Upstate (Temecula, CA). FITC-conjugated goat anti-mouse IgG1 and PE-conjugated goat anti-rabbit IgG was distributed by Southern Biotech (Birmingham, AL). Polyclonal MET, RAS, p21 and tubulin antibodies were distributed by Santa Cruz Biotechnology (C-12, FL-189, SC-397 and h-235 clones, Santa Cruz, CA).

RNA Interference. The sequences of the siRNA in the CDT2 specific ON-TARGET *plus* SMART pool are as follows: 3'-GUCAAGACCUGGCCUAGUA-5'; 3'-GAAUUAUACUGCUUAUCGA-5'; 3'-ACUCCUACGUUCUCUAUUA-5'; 3'-GCGCUUGAAUAGAGGCU UA-5'.

RNA extraction and Quantitative Reverse Transcription-PCR.
Total RNA was extracted and purified using the SV Total RNA Isolation System (Promega, Madison, WI) as suggested by the manufacturer. RNAs were then quantified and inspected by Bioanalyzer (Agilent Technologies, Waldbrom, Germany) analysis. cDNA was synthesized from 500 ng RNA using MMLV-RT(H-) enzyme (Promega, Madison, WI). Quantitative reverse transcription-PCR was done on an MyiQ Thermal Cycler (Bio-Rad, Hercules, CA); all quantitative PCR mixtures contained 20 ng retro-transcribed RNA, 1x SYBR Green PCR Master Mix (2x; Applera, Foster City, CA), and 300 µM of each target specific primer. Each target genes expression was evaluated using a relative quantification approach $2^{-\Delta\Delta CT}$ method with cyclophilin A (PPIA, NM_021130) as internal reference. Primer sets and PCR cycling conditions are available from the Authors.

Flow cytometry analysis. Cell cycle analysis was based on DNA content. Ethanol-fixed cells were treated with 1 µg/ml RNase A (Saint Louis, MO) and stained with 50 µg/ml PI. Nuclei were analyzed using the CyAn (DakoCytomation) and cell cycle distributions were plotted with Summit v4.3 software (DakoCytomation). For immunofluorescent detection of phosphorylated histone H3 the cells were harvested, washed with phosphate-buffered saline (PBS), fixed in cold 70% ethanol and stored at -20°C for up to 2 weeks before analysis. After fixation, the cells were washed with Tris-buffered saline pH 7.4 (TBS) and then rehydrated for 10 min at 4°C in TBS containing 4% FBS and 0.1% Triton X-100 (TST) (Sigma-Aldrich Co., St Louis, MO). After centrifugation, the cell pellet was suspended in 50 µl of TST containing anti-phosho-Histone H3 (Ser10) (Upstate, Temecula, CA) diluted 1:100 in TST and incubated for 2 h at 37°C. After two washes with TBS, they were resuspended in FITC-conjugated goat anti-mouse IgG1 (Southern Biotech, Birmingham, AL) diluted

1:50 in TST as secondary antibody, and shaken for 1 h at room temperature in the dark. Next, they were washed with TBS and their phase in the cell cycle was determined from their DNA content.

Supplementary Table 1: Details of tissues of origin and mutations of cell lines

cell lines	organ	histology	tumorigenesis	TP53 mutation	other mutations
A549	lung	non-small cell carcinoma	YES	WT	CDKN2A, CDKN2a(p14), KRAS, SMARCA4, STK11(LBK1)
DLD-1	colon	adenocarcinoma	YES	MUT	KRAS PIK3CA
EBC-1	lung	squamous cell carcinoma	YES	MUT	MET (amplification)
HCT-116	colon	carcinoma	YES	WT	CDN2A, CDKN2a(p14), CTNNB1, KRAS, MLH1, PIK3CA,
HeLa	cervix uteri	adenocarcinoma	YES	WT downregulated HPV+ (18)	STK11 (LKB1)
HOS	bone	osteosarcoma	YES	MUT	CDKN2A, CDKN2a(p14)
HS 746T	stomach	gastric carcinoma	YES	WT	MET mutation and MET amplification
MG-63	bone	osteosarcoma	YES	MUT	CDKN2A, CDKN2a(p14), MYC (amplification)
SK-OV-3	ovary	adenocarcinoma	YES	MUT	CDKN2A, CDKN2a(p14), PIK3CA, MLH1, ERBB2 (amplification)
Suit-2	pancreas	Cancer	YES	MUT	KRAS, CDKN2A-B (p16)
TOV-21G	ovary	clear cell carcinoma	YES	WT	KRAS2, TGFb-RIIc
U-2 OS	bone	osteosarcoma	YES	WT	
HK2	kidney	proximal tubule	No	WT downregulated HPV+ (16)	
hTERT-HME1	breast	mammary gland	No	MUT	
MCF 10A	breast	mammary gland	No	WT	MYC, CDKN2A-B (p16)
MRC-5	lung	fibroblast	No	WT	

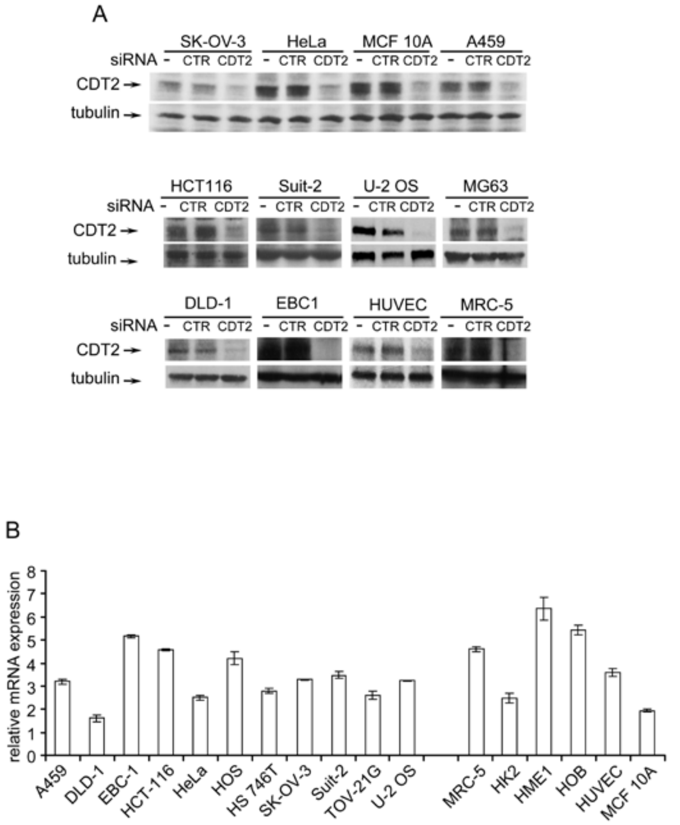

Supplementary Figure 1

Expression and silencing of CDT2 in cancer cell lines and in non-transformed cells. A, Western blot analysis of CDT2 protein expression in cell lines transfected with either the CDT2 specific siRNA (CDT2) or control siRNA pool (CTR) after 72 hours. B, Quantitative PCR of CDT2 mRNA in cancer cell lines (grouped on the left) and non-transformed cell lines (grouped on the right).

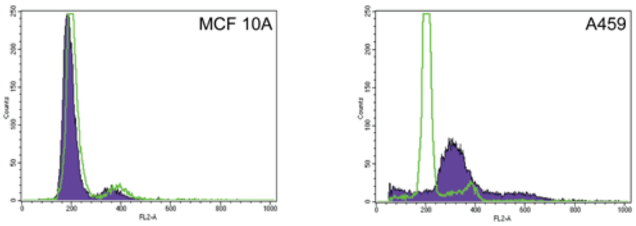

Supplementary Figure 2
Stable CDT2 suppression with CDT2 specific shRNA affects viability of the A549 cancer cells (on the right) but not that of the non-transformed MFC10A cells (on the left). Cell lines were transduced to express CDT2 specific shRNA (purple) or control shRNA (green line). Percentage of cells was measured with cytometry after cell labelling with propidium iodide. Only A549 cells accumulated in sub G0/G1 after CDT2 suppression, i.e. underwent apoptotic death.

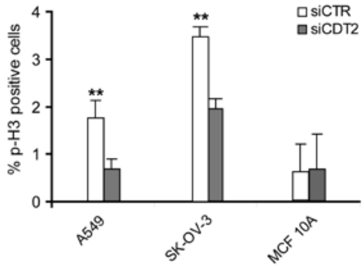

Supplementary Figure 3
CDT2 suppression resulted in decreased histone H3 phosphorylation in cancer cells, but not in non-transformed cells. Quantification of cells showing histone H3 Ser10 phosphorylation (pH3) by means of cell labeling with the phospho-specific antibody and cytometric analysis. In A549 and SK-OV-3 cancer cells CDT2 depletion caused a decrease of the pH3 positive cells. In the non-transformed MCF 10A cells, CDT2 depletion did not change cell staining with the anti-pH3 antibody; significance was evaluated using the Student's t-test: ** P<0.01.

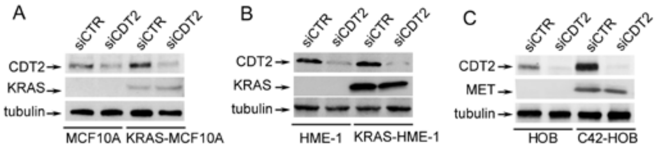

Supplementary Figure 4
Expression and silencing of CDT2 in non-transformed cells made transformed by the addition of a hyper-activated oncogene, analyzed with Western blot analysis. A, KRAS and CDT2 protein expression in the non transformed, spontaneously immortalized MCF 10A breast epithelial cells and in the same cells made transformed and tumorigenic by the addition of the activated KRAS (KRAS-MCF 10A). B, KRAS and CDT2 protein expression in the non transformed, hTERT immortalized HME-1 breast epithelial cells and in the same cells made transformed and tumorigenic by the addition of activated KRAS (KRAS-HME-1). C, MET and CDT2 protein expression in human osteoblasts (HOB) and in the same osteoblasts made transformed and tumorigenic by the over-expression of the MET oncogene obtained by transducing cells with a Lentiviral vector and integrating multiple copies of the oncogene (C42-HOB).
